# Supplementary material for: A Global Screen for Assembly State Changes of the Mitotic Proteome by SEC-SWATH-MS
Source: Cell Syst. 2020 Feb 26;10(2):133–155.e6. doi: 10.1016/j.cels.2020.01.001 (PMC7042714; doi:10.1016/j.cels.2020.01.001)

Q8IXQ4 | GPAM1_HUMAN | GPALPP1 KIAA1704 LSR7 AD029
Monomer MW [kDa]: 38.142 Monomer expected elution fraction: 49

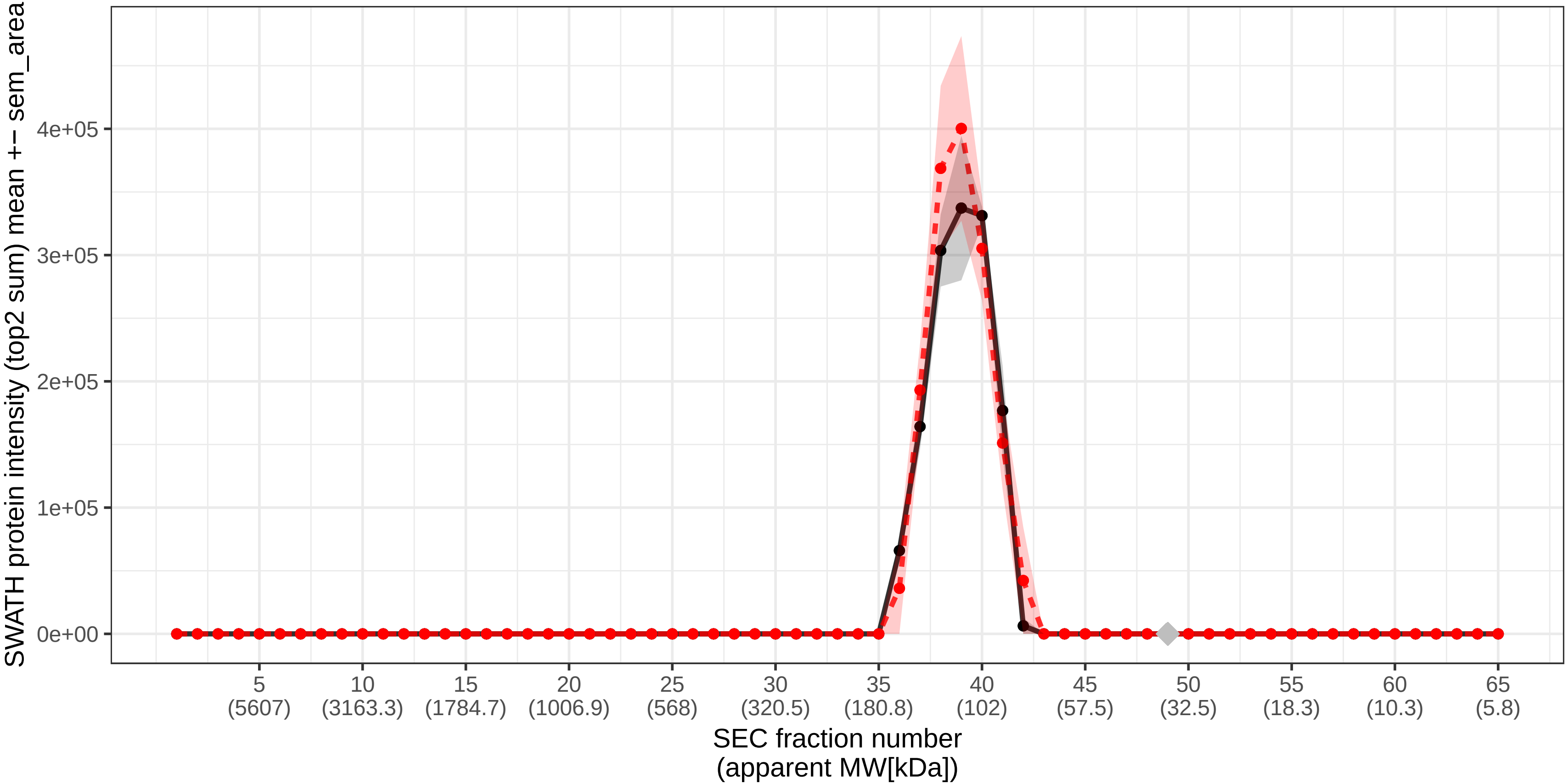

Supplement: Data S1. SEC-SWATH-MS Protein Chromatograms, Related to Figure 1 [file mmc6.zip › SECchrom_Q8IXQ4_GPAM1_HUMAN_GPALPP1_KIAA1704_LSR7_AD029.pdf]
